# Self-organization of waves and pulse trains by molecular motors in cellular protrusions

**DOI:** 10.1038/srep13521

**Published:** 2015-09-03

**Authors:** A. Yochelis, S. Ebrahim, B. Millis, R. Cui, B. Kachar, M. Naoz, N. S. Gov

**Affiliations:** 1 Department of Solar Energy and Environmental Physics, Swiss Institute for Dryland Environmental and Energy Research, Blaustein Institutes for Desert Research (BIDR), Ben-Gurion University of the Negev, Sede Boqer Campus, Midreshet Ben-Gurion 84990, Israel; 2 Laboratory of Cell Structure and Dynamics, NIDCD, National Institutes of Health, 50 South Drive, Bethesda, MD 20892-8027, USA; 3 Department of Chemical Physics, Weizmann Institute of Science, P.O.B. 26, Rehovot, Israel 76100

## Abstract

Actin-based cellular protrusions are an ubiquitous feature of cells, performing a variety of critical functions ranging from cell-cell communication to cell motility. The formation and maintenance of these protrusions relies on the transport of proteins via myosin motors, to the protrusion tip. While tip-directed motion leads to accumulation of motors (and their molecular cargo) at the protrusion tip, it is observed that motors also form rearward moving, periodic and isolated aggregates. The origins and mechanisms of these aggregates, and whether they are important for the recycling of motors, remain open puzzles. Motivated by novel myosin-XV experiments, a mass conserving reaction-diffusion-advection model is proposed. The model incorporates a non-linear cooperative interaction between motors, which converts them between an active and an inactive state. Specifically, the type of aggregate formed (traveling waves or pulse-trains) is linked to the kinetics of motors at the protrusion tip which is introduced by a boundary condition. These pattern selection mechanisms are found not only to qualitatively agree with empirical observations but open new vistas to the transport phenomena by molecular motors in general.

Actin-based cellular protrusions are important to variety of biological processes, ranging from cell migration and cell morphology to cellular communication^1^ and morphogenesis^2^. In fact, many functionalities of living cells depend on a complex transport that is manifested mostly by a variety of molecular motors that processively move along the actin filaments^3^. Unconventional myosins (UM) constitute arguably the most important family of processive motors, moving and transporting numerous cargos towards the plus-ends (growing tips) of actin-filaments (except for myosin-VI which is a minus-end directed motor). Inside actin-filled cellular protrusions, such as filopodia, the actin filaments are cross-linked, and form a bundle that is polar: while (most) of the plus-ends are at the filopodia tip since the filaments are not continuous from the base to the tip there may also be plus (and minus) ends present throughout the length^4^. Actin-filled protrusions serve a variety of roles in biology: filopodia are instrumental for cell motility and cell-cell communication^5^, while stereocilia constitute the final mechanical element in the hearing process^6^. UM cargos can be viewed as chemical information, i.e. key proteins which play an essential role in controlling the rate of actin polymerization at the filaments’ tips, and the organization of the actin cytoskeleton^7^. Examples of such UM cargos include Mena/Wasp^8^, Whirlin^9^ and Espin^10^. Accordingly, suppression or mutation of UM often results in dysfunctional behavior and/or morphology of actin-based structures in the cell, such as absence of filopodia^11^ or abnormal stereocilia^12^. Thus, understanding the dynamics of UM motors is central to the understanding of actin dynamics and thus the dynamics of the numerous actin-based structures in cells, specifically actin-based cellular protrusions such as filopodia^5^ and stereocilia^13^.

Intriguingly, analyzing the dynamics of UM within actin protrusions demonstrates several distinct macroscopic spatiotemporal behaviors, i.e. spatially extended concentrations of UM that are significantly above a single molecule scale. The first type of behavior is related to *tip accumulation*: *In-vivo* experiments show that tip-directed myosins exhibit an accumulation at the protrusion tip^13^ (myosin-III in Fig. 1). In stereocilia, for example, some UM are highly localized at the tip (myosin-XV), while others are spread over a large region below the tip (myosin-III)^13^. What determines these different profiles, especially when there is coexistence of several types of UM within the same protrusion^14^, is a fundamental open question. The second type of behavior is related to self-organization into *quasi-periodic* (hereafter traveling waves) or *isolated aggregates* (hereafter pulses) that propagate towards the protrusion base (myosin-XV in Fig. 1). Propagating aggregates are observed in both filopodia and stereocilia by myosin-X^15^,^16^,^17^,^18^, myosin-XV^19^, myosin-III^10^ and myosin-Va^17^. Notably, molecular transport has been explored in the context of molecular motors on microtubules (MT) which occur in open-system conditions, with a large surrounding reservoir available for exchange^20^,^21^, a situation that is not applicable to UM inside protrusions. While UM aggregations are thought to form a recycling mechanism that enables myosins to bring new cargo from the bulk, their nature and underlying mechanisms are unknown, for example does this complex behavior stem from stochastic or deterministic origin?

Here we use a mass conserving reaction-diffusion-advection framework to unfold mechanistically the origin of self-organized UM aggregates. We consider three main modes of transport for molecular motors within the actin-filled protrusions: free diffusion when not bound to the actin filaments, processively walking to the protrusion tip when bound to the actin, and advected towards the protrusion base when bound the actin but in a non-processive (stalled) mode. The motors are considered to have the minimal set of reactions that describe the transitions between these three states. To model the system, we consider a minimal form of non-linear interactions among UM, via transition rates, and account for the dominant forms of transport fluxes, namely processive transport, free diffusion and advection by the treadmilling actin. In particular, the model results reveal the coexistence of two families of large amplitude, self-organized aggregates within the protrusion: (*i*) traveling waves (TWs) and (*ii*) pulse trains. While in reality the two families may be indistinguishable apriori, we find that the selection between these two patterns is dominated and can be traced by the boundary conditions at the protrusion tip, specifically whether UM accumulate on the actin bundle or fall off. The model results have been found to qualitatively agree well with experimental observations and thus, suggest novel insights into the link between dynamic mechanisms that take place inside cellular protrusions.

## Results

### Formation of Myosin aggregates at the Filopodia tip: Experiments

**Figure 1:**
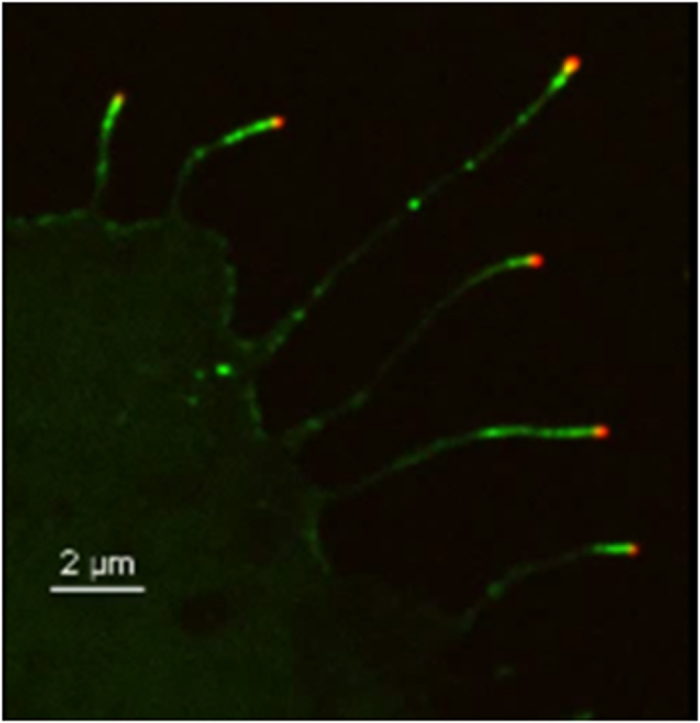
Image of several filopodia in COS7 cells (Supporting Movie S1), with myosin-XV labelled fluorescently in green and myosin-III labeled in red. Myosin-XV aggregates form at the tip and move rearwards from the tip, at roughly the actin treadmilling velocity, while myosin-III aggregates remain localized at the tip.

**Figure 2:**
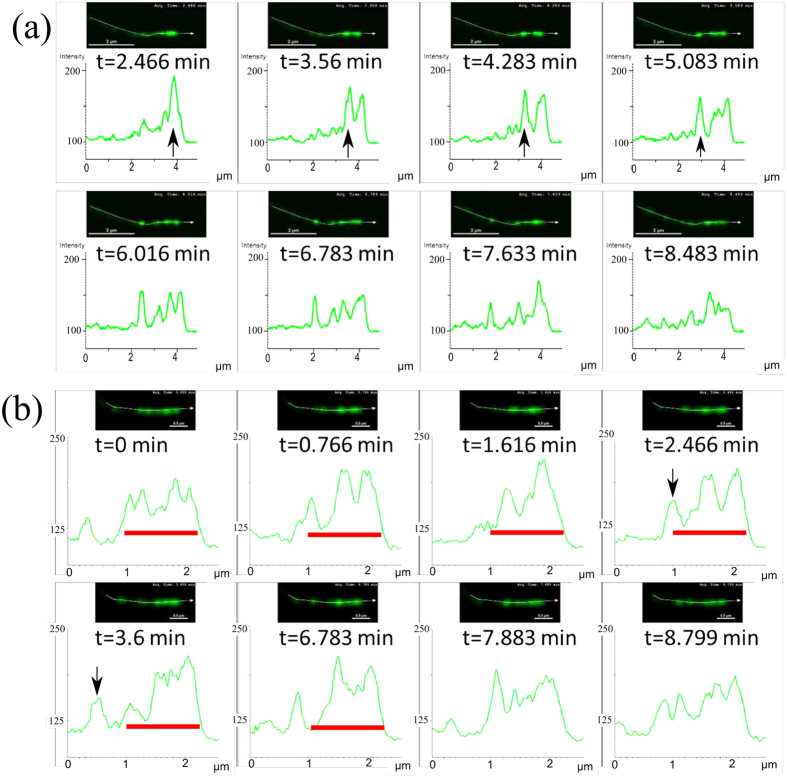
Temporal snapshots of Myosin-XV inside relatively short filopodia (<5 *μm*) at different times, with Myosin-XV labelled fluorescently in green (top panels), and respective concentration profiles at the bottom. In (**a**) aggregates that form at the tip (black arrow) and move rearwards from the tip, in the form of isolated pulses and pulse trains. In (**b**) the tip region gets “filled” with motors (indicated by the red bar), develops undulations (TWs), and eventually initiates release of pulses (black arrow).

**Figure 3:**
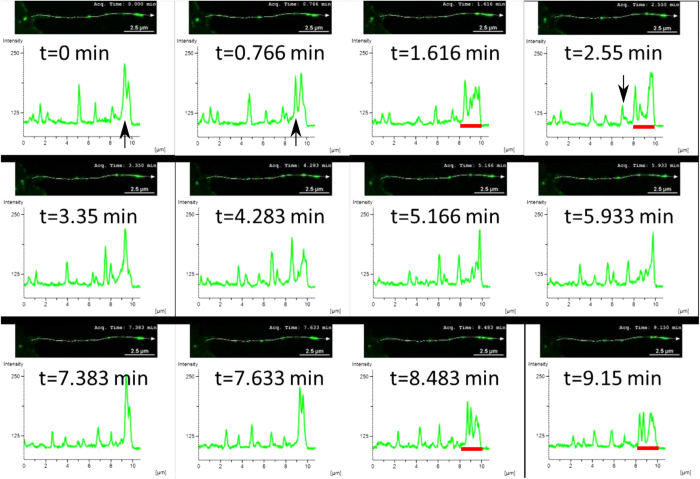
As in Fig. 2, but for relatively long filopodia (~10 *μm*). Pulses that originate at the tip are indicated by black arrows, and the cases showing tip-accumulation mechanism are indicated by red bar.

To investigate efficiently the dynamics of the formation and propagation of aggregates (which is absent in Fig. 1), we collected new data of live imaging of mysoin-III and myosin-VX motors within filopodia at high spatio-temporal resolution (for a detailed description see the Experimental Methods section). Typical profiles of myosin-XV distributions in filopodia are shown at distinct times in Figs 2 and 3. Notably, the high concentrations (pulses) are at about the optical resolution limit. The following observations are found of main importance:

- Unlike the stationary profiles of myosin-III (indicated by red in Fig. 1), myosin-XV motors appear to accumulate at the flopodia tip, but this accumulation is highly variable and changes over time.
- At the tip, accumulation of myosin-XV can result in two types of behavior: (i) Immediate breakdown into isolated aggregates (Fig. 2a), or (ii) first, “filling-up” rather uniformly a finite region that extends towards the bulk and then break up of this domain into undulations that propagate towards the filopodia base (Fig. 2b).
- Both mechanisms appear to be independent of the filopodia length, i.e., the mechanisms are observed in both short and long filopodia. In long filopodia the width of the aggregates is much smaller than the filopodia length, as shown in Fig. 3.
- The aggregates tend to diminish in their amplitude as they propagate towards the filopodia base, as shown in Fig. 2 and Supplementary Fig. S1b.
- By examining the empirical results, it is difficult to correlate between the shape of the aggregates that propagate through the filopodia (from tip to base) and their formation mechanism at the tip, i.e. TWs resemble the pulse trains and vise-versa (Fig. 2).
- The lack of any significant entrainment of myosin-III by the myosin-XV aggregates (Fig. 1) implies weak interactions among them, and negligible excluded-volume effects along the filopodia.

Consequently, these observations suggest two novel dynamical modes by which the tip accumulation becomes unstable and produces propagating aggregates. In what follows, the two observed initiation mechanisms will be both qualitatively identified and compared to the results of a theoretical model that exhibits behavior of this type. Moreover, we will show that focusing on the selection origin at tip allows to effectively distinguish between two distinct transport mechanisms, i.e., transport by TWs or by pulses. Our work is the first to treat this phenomenon using a continuum model, which can be compared to a discrete model proposed recently^22^. In order to correspond to the experiments we use in the calculations the actin treadmilling velocity observed in the filopodia: 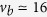 nm/sec. Since there are no single-molecule measurements of the myosin-XV velocity, we use in the simulations the velocity measured for myosin-X (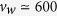 nm/sec^17^), since they share structural similarity and are both highly processive.

### Construction of the theoretical approach

**Figure 4:**
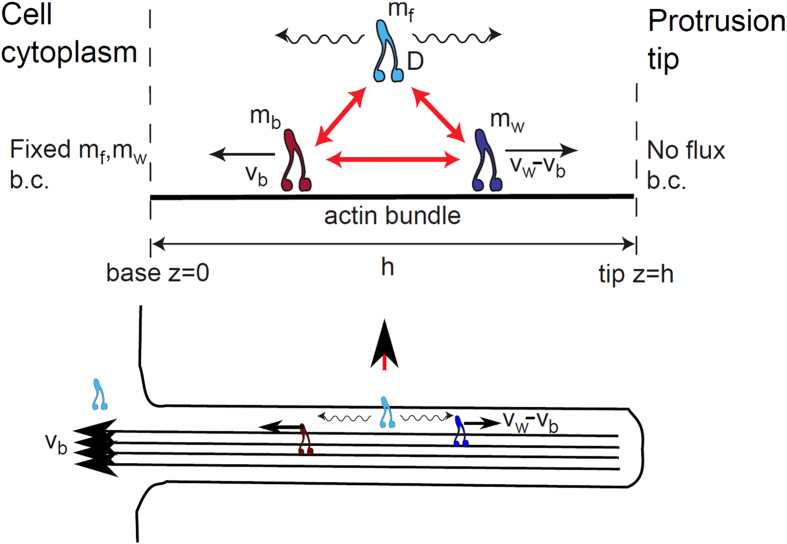
Illustration of a one-dimensional model of motors inside a linear protrusion, moving along unipolar filaments. Free motors (light blue) diffuse (with diffusion coefficient *D*), bound motors (red) drift towards the protrusion base at the treadmilling velocity *v*_*b*_, and processively walking motors (blue) with velocity *v*_*w*_ − *v*_*b*_ towards the tip. Reversible transitions are assumed between all states (red arrows).

Inside the protrusion, we consider three generic mean-field forms of UM fluxes (Fig. 4): (i) processive motor current (to the tip, except for the base-directed myosin-VI), *m*_*w*_ (ii) motors that relate to the treadmilling actin flow (to the base), *m*_*b*_ and (iii) free diffusion of motors (motors that are not attached to actin filaments), *m*_*f*_. Although other motor types can be also involved, each one can only experience these three modes of transport (migration and/or diffusion). This approach is the simplest form that contains an internal degree of freedom of the motors which affects their processivity, i.e. affects the average velocity along the actin^14^. The above description can be viewed as a simplified realization of complex chemical steps involved in motor activity or inactivation (becoming less or non-processive). For example, it was found that several types of molecular motors become processive only when they are bound to a cargo molecule^14^,^23^. In many cases, the cargo is involved in regulating the actin polymerization at the protrusion tip^8^,^10^,^12^, where it normally detaches from the motor, which subsequently becomes inactive after release of the cargo. When inactive, motors may detach from the actin filament and diffuse freely, or stay attached^23^ and drift towards the protrusion base due to the actin treadmilling. Furthermore, neighboring motors can “steal” the cargo^14^, thereby inactivating each other. There are other forms of interactions among motors that result in their inactivation, for example the auto and trans-phosphorylation among myosin-III motors^24^,^25^. Therefore, it is applicable to treat the motor self-organization as a *coarse-grained* description of a complex set of underlying interactions in which the three main species under consideration: processive, stalled (non-processive) and diffusive, correspond to the observed dynamics of motors in protrusions^17^. Consequently, irrespective of the particular biochemical details of different motor species, our model captures the general forms of motor transport inside the protrusion.

In addition, molecular motors may exhibit local interactions/transitions between the different motor species as they move along the protrusion. Previous studies have modeled transitions from one motor state to another by first order (linear) kinetics^26^. However, the latter cannot support self-organization in the form of nonlinear TWs and pulses^27^,^28^, and we therefore propose an additional higher order transition rates for the transition from the processive to the stalled state. This autocatalytic (positive feedback) non-linearity may correspond for example to a cooperativity effect, where the inactivation transition rate is enhanced by the simultaneous interactions of several neighboring motors (and possibly also their cargos). This is similar to the observed increase in phosphorylation among myosin-III motors at high concentrations^24^,^25^, which reduces their processivity significantly.

Actin-based cellular protrusions are complex and heterogeneous media and yet the geometrical properties can be simplified to allow a continuum mean-field description. Consider a uniform cylindrical protrusion of constant height and actin polymerization rate, filled with actin filaments whose polymerizing (barbed) ends are at the protrusion tip. In addition, assume that all the actin filaments extend from the protrusion tip to the cytoplasm at the protrusion base. Under these simplifications, we can coarse-grain the description of the motors within the protrusion, as depicted in Fig. 4. Furthermore, we project the volume within the protrusion to a one-dimensional representation, where the distribution of myosin motors varies only along the protrusion axial coordinate *z*, and is uniform within each cross-section^26^. In particular, if the actin filaments are strongly cross-linked into a bundle, the motors can move only within the annular space between the actin core and the surrounding membrane.

With respect to the local transition rates, the continuum description always admits coexistence between stalled and processive motors at the same location *z* along the protrusion. While this is impossible microscopically for a strictly one dimensional track^22^, our one dimensional space effectively represents all the actin filaments at the surface of the bundle inside the protrusion, whereby hopping between multiple tracks allows the “tunneling” of processive motors through a region that is rich in stalled motors. Furthermore, at present it is unknown if the observed aggregates span the entire bundle surface, or form on just several (or even one) actin filaments.

In what follows, we identify model parameters such as diffusion coefficient, propagation speed, transition rates by 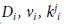, respectively; formulation and details of the theoretical model are given in detail in the Methods section.

### Model results

We describe here the main theoretical results regarding the dynamics of the motors along the protrusion. Due to mass conservation, a key property of the model (Eq. 1, 2, 3) is an infinite coexistence of uniform solutions on unbounded domains, which is broken by the boundary conditions (BCs). Typical biological conditions in the cell bulk dictate effectively the BC at the filopodia base that correspond to a fixed (and low) concentration of the myosin motors. In what follows, this condition yields a uniform steady-state solution of low concentration, hereafter we referred to it as *M*^*L*^, which is stable for typical values (for details see Section *Uniform solutions and linear stability analysis* in Methods). The other boundary condition, at the filopodia tip, corresponds to the zero-flux constraint (Eqs. 5,9), which gives for typical values of the parameters a uniform steady-state solution with high concentration of bound motors, hereafter referred to as *M*^*U*^. Unlike the *M*^*L*^ state, the *M*^*U*^ state is unstable to TWs (for details see Section *Uniform solutions and linear stability analysis* in Methods). We show next, that the effective existence of this bistability and the instability of *M*^*U*^ are responsible for the rich dynamics.

#### Stationary aggregation at the tip

**Figure 5:**
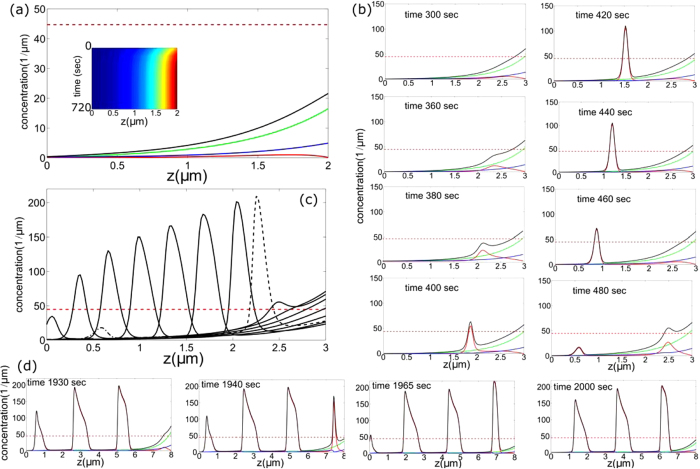
(**a**) Steady-state concentration profiles for domains under a critical length (*h*<*h*_c_) and *β* = 0. The inset shows a corresponding space-time plot of the total myosin concentration. The concentrations of the stalled, processive and free motors (*m*_*b*_, *m*_*w*_, *m*_*f*_) are denoted by the red, blue and green lines respectively, and the sum total concentration in black. The horizontal dashed red line denotes the concentration of stalled myosins in the *M*^*U*^ state. Parameters used are: *v*_*w*_ = 0.6 *μ*m/sec, *v*_*b*_ = 0.016 *μ*m/sec, *D* = 0.1 *μ*m^2^/sec, *D*_*b*_ = 0.0001 *μ*m^2^/sec, 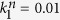
sec^−1^, 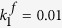
sec^−1^, 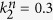
sec^−1^, 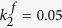
sec^−1^, 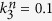
sec^−1^, 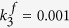
sec^−1^, *k*_*bw*_ = 2 *μ*m^2^ and 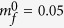 *μ*m^−1^. (**b**) Snapshots of dynamics on large domains *h* > *h*_*c*_ in which pulses form, and propagate towards the protrusion base. The pulses are generated periodically. (**c**) Overlayed time course (at 30 sec intervals) of a single pulse (solid line) that forms and decays as it propagates (dashed-dot and dashed pulses are from the previous and next cycles respectively). (**d**) Pulse train in a longer protrusion.

Before describing propagating aggregates, we begin by describing stationary accumulations of UM at the tip region, which are also often observed in experiments^13^ (myosin-III, red labeling in Fig. 1). In our model this happens when the accumulation of motors at the tip is balanced by the diffusion current from the tip to the base, and the accumulation does not come close to the unstable *M*^*U*^ state (dashed-dot line in Fig. 5a and Eqs. 7, 8, 9). When the conversion from processive to stalled is low, the UM distributions closely resemble the *m*_*b*_ → 0 limit described in^26^, preventing any nonlinear spatiotemporal dynamics. This condition occurs for the case of motors falling-off at the tip (*β* = 0), and a protrusion that is below a critical height *h* < *h*_*c*_ (Fig. 5a), such that the accumulation of stalled motors at the tip results in a small perturbation; *h*_*c*_ depends on the model parameters (Eqs. 1, 2, 3) such that: 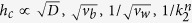, since this length-scale is essentially given by the solution of the non-interacting system^26^. Note that the stable accumulation at the tip will appear for UM of low processivity and high actin retrograde flow. In Fig. 5a we demonstrate that for myosin-XV-like parameters the stationary accumulation is achieved for very short filopodia: we find 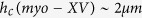, for the parameters used in our simulation. For myosin-IIIa we use an upper-bound on the velocity, estimated from the measured ATPase rate^14^: 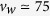 nm/sec. We therefore expect 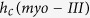 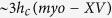, but in the experiments the stationary myosin-III accumulation persists even for the longest filopodia (>10 *μm*), suggesting that for this motor the non-linear conversion parameter *k*_*bw*_ may be much lower as well (Eqs. 2,3). The case of myosin-III with *β* = 1 at the tip is given in Supplementary Fig. S4, showing the protrusion gets filled by motors, unlike the observation of tip accumulation (Fig. 1). Note that since we do not know the exact rate parameters for the motors, we do not attempt to make a quantitative fit to the data, but rather compare the generic qualitative dynamics of the model to the experiments. Weakly processive motors, such as myosin-III^10^, may be stable at the tip of filopodia with fast actin treadmilling, but form aggregates when the actin polymerization rate is low (such as in stereocilia), since the critical height 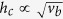.

#### Pulses and pulse trains

In the case where the flux of processive motors is converted to freely diffusing at the tip (*β* = 0), we obtain at the tip 
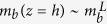
, as confirmed by Fig. 5a. Consequently, most of the domain resides in the linearly stable *M*^*L*^ state and any accumulation at the tip, is regarded as a spatially localized *large* amplitude perturbation. Notably, this behavior cannot be deduced from a linear theory and is related to properties of global bifurcations^29^. Due to unbalanced diffusion current from the tip to the base, for *h* > *h*_*c*_, accumulation of motors increases and there exists a critical threshold for which propagating pulses form, as shown in Fig. 5b. Note that the pulses form periodically, as the accumulation near the tip is depleted, and then builds up after each pulse is released. The first pulses that form at the tip decay fast in amplitude (by preserving the overall width) as they propagate towards the protrusion base (i.e. for low values of *M*^*L*^), but over time the overall background values of the motors along the protrusion length increase and the pulses decay more slowly (Fig. 5c). This behavior is more profound on larger domains where excitable-type pulses indeed form and reach (after a finite time) the filopodia base, as shown in Fig. 5d and Supplementary Fig. S3.

In the biological context, the pulses are a form of self-organized traffic-jams of motors, whereby processive motors continuously join the aggregate from the protrusion base, and leave it from the other side towards the tip. As the processive motors hit the aggregate, the local high concentration of stalled motors induces their non-linear conversion to the stalled mode. This is similar to motors getting stopped by a traffic-jam of non-processive motors blocking their way on the actin tracks. A similar phenomenon occurs for example in vehicle traffic, where the high density region of the jam propagates due to the influx of vehicles at one end of the jam, and their release from its opposite end^30^. In our model the non-linear conversion term reflects a similar effect.

#### Traveling waves

**Figure 6:**
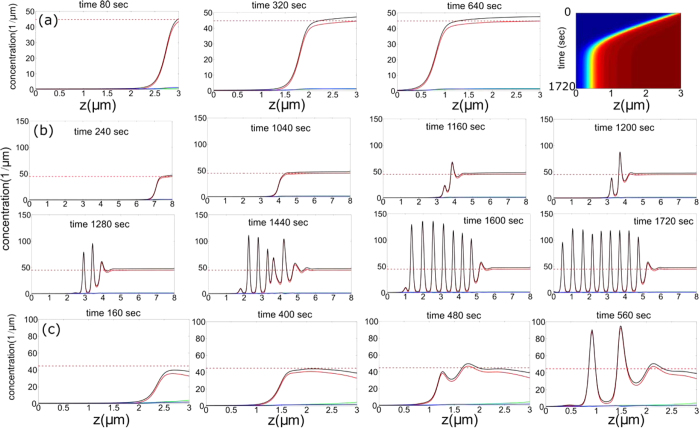
(**a**) Concentration profiles of with BC at the tip *β* = 1. Space-time plot of the total myosin concentration is in the right-most panel. Color scheme and parameters as in Fig. 5. For convection and short protrusion (*h*<*h*_c_) a quiescent state is obtained, where the unstable modes that initiate the TWs are washed out before they have time to grow. (**b**) Snapshots showing the evolution of unstable modes into TWs on larger domains (*h* > *h*_*c*_). (**c**) Snapshots for BC at the tip *β* = 0.5.

**Figure 7:**
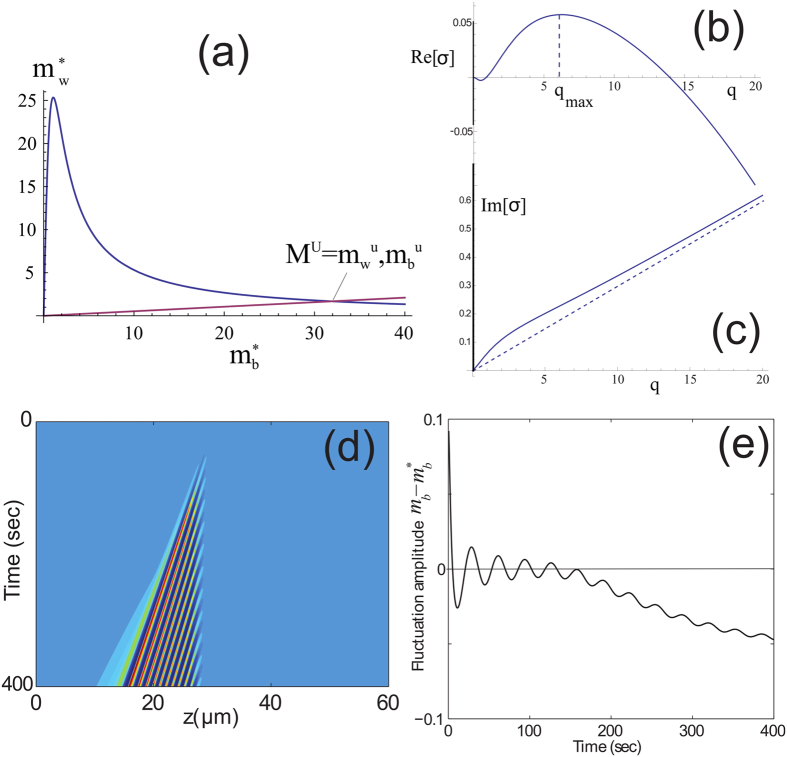
(**a**) Continuum of uniform steady-state solutions, for 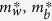 (Eqs.(1), blue line). The purple line indicates the zero-flux condition which determines *M*^*U*^. (**b,c**) Real and imaginary parts (respectively) of the dispersion relation about *M*^*U*^. The most unstable mode *q*_*max*_ is denoted in (**b**), while the advection velocity *v*_*b*_ is denoted by the dashed line in (**c**). (**d**) Space-time plot of the fluctuation amplitude 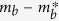 after an initial perturbation located at *z* = 30 *μ*m, where 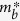 corresponds to the *M*^*U*^ state. (**e**) Exponential decay of the fluctuation in time at the initiation site (in addition to a shift of the overall background due to the spreading of the initial perturbation). Parameters used are: *v*_*w*_ = 0.6 *μ*m/sec, *v*_*b*_ = 0.03 *μ*m/sec, *D* = 0.1 *μ*m^2^/sec, *D*_*b*_ = 0.0005 *μ*m^2^/sec, 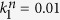
sec^−1^, 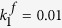
sec^−1^, 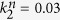
sec^−1^, 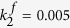
sec^−1^, 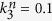
sec^−1^, 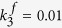
sec^−1^, and *k*_*bw*_ = 2 *μ*m^2^.

**Figure 8:**
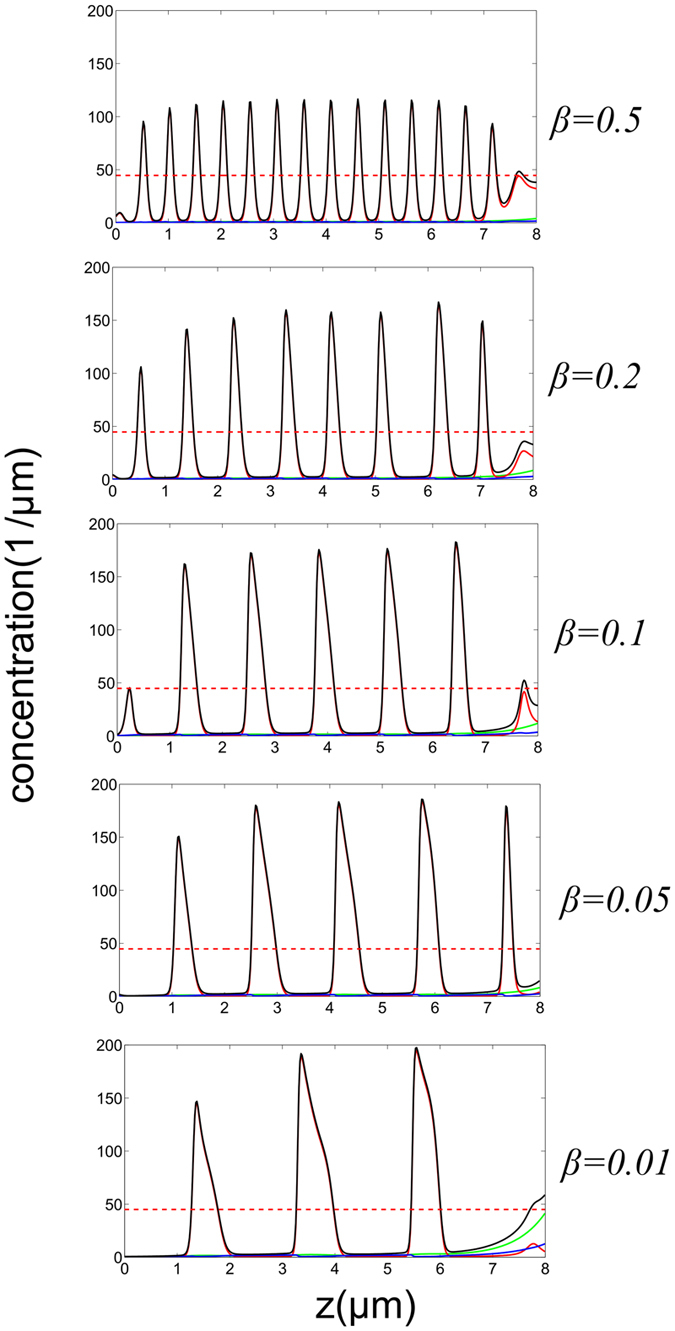
Comparison between concentration profiles that are obtained under distinct conversion rates at the tip, i.e., *β* = 0.5, 0.2, 0.1, 0.05, 0.01 from top to bottom, respectively.

The emergence mechanism of TWs can be understood from the instability of *M*^*U*^ which is ultimately enforced by the high conversion at the tip between the counter propagating motors, i.e., *β* = 1 (Eq. 6). Initially the domain is at the *M*^*L*^, however after certain time a connection (hereafter front) between the *M*^*L*^ and *M*^*U*^ states is formed: at the protrusion base (*z* = 0) the fixed cytoplasm concentration selects the *M*^*L*^ state, while at *z* = *h* the choice of *β* = 1 selects the *M*^*U*^ state. For large enough values of *v*_*b*_, the front solutions that connect bi-asymptotically *M*^*L*^(*z* → 0) and *M*^*U*^(*z* → *h*) propagate in a way that the *M*^*U*^ state invades the *M*^*L*^ state (Fig. 6a), while its width is determined by diffusion. In this case, the instability about *M*^*U*^ develops at a much slower rate than the drifting velocity, i.e., TWs cannot develop before the front reaches the protrusion base. Fig. 6a shows the formation of a stationary (quiescent) state while the convective nature of the instability is shown in Fig. 7. For lower *v*_*b*_ values or longer protrusions the growth rate of the most unstable *M*^*U*^ mode allows the formation of TWs at the front region, and propagate from the front region towards the base, as shown in Fig. 6b. The triggered TWs correspond to a wavelength of the most unstable mode *q*_*max*_ found in the linear stability analysis (Fig. 7b), while non-linearities modify the wavelength when the amplitude is large. For mixed BCs, 0 < *β* < 1, the behavior is intermediate in between the TWs and pulses, as shown in Fig. 8. This is explained as following: Proximity of *β* to 1 allows access to the basin of attraction of TWs that emanate from *M*^*U*^ (and other degenerate unstable uniform states), while low *β* values are related to stable uniform states and thus allow formation of pulses. A similar behavior also applies to short filopodia, as demonstrated in Fig. 6c.

## Discussion: comparing between the model and experiments

**Figure 9:**
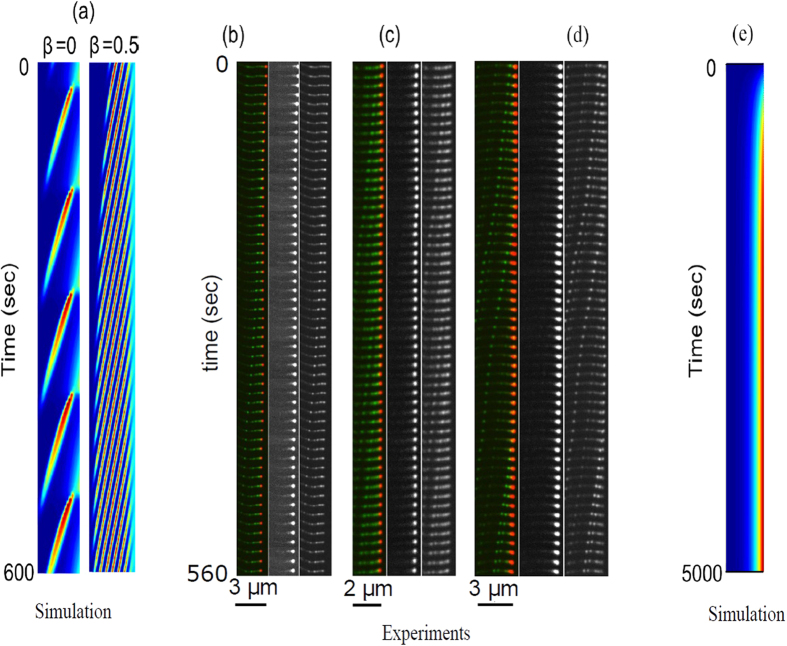
(**a**) Space-time plots of the model simulations for total concentration, with parameters as in Fig. 5, for different values of *β* (*h* = 3 *μ*m). (**b–d**) Kymographs showing motors in filopodia in experiments on COS7 cells, with the green label for myosin-XV and the red label for myosin-III (left panels). Middle/right panels show myosin-III/XV, respectively. Filopodia lengths in the experiments are around 2–3 *μ*m. In some cases we observe high frequency of pulses (**b,c**), corresponding to larger values of *β* (see in (a)), while in others we find a lower frequency (**d**), which corresponds to a lower value of *β* → 0. (**e**) Space-time plot for the total concentration of slow motors, such as myosin-III (*v*_*w*_ = 0.075 *μ*m/sec), demonstrating formation of a stable concentration accumulation at the tip.

An advantage of the employed theoretical approach is that it undertakes the phenomenological principles of pattern selection mechanisms in a similar fashion that is frequently performed in chemical and biological systems, on the other hand this approach naturally limits quantitative comparisons^27^. Comparing the simulation results with the experimental observations (Fig. 9), we find a very good qualitative correspondence between the interrelation of pulses and TWs and the triggering at the protrusion tip. We emphasize that these qualitative pattern selection features are rather general and are anticipated to persist also once the model equations are modified to describe more realistic behavior. Notably, the observed dynamics do not ultimately correspond to either of the *β* = 0,1 cases, indicating that the actual nature of the boundary condition (value of *β*) at the tip may fluctuate between these two limiting behaviors. The model simulations demonstrate that for *β* → 0 (*β* → 1) pulses (TWs) emerge and can be distinguished by their periodicity, and the width of the accumulation and its depletions at the tip (Figs 5, 6, 7, 8, 9). Note that while for both *β* = 0,1 there are pulses propagating to the filopodia base, there is a big difference in frequency (Fig. 9a). The experimental data shows a mixture of both high and low frequency of aggregates (Fig. 9b–d), indicating the variability in *β*.

Using the BC at the tip of motors falling off (*β* = 0), we find that the strong aggregates that form for myosin-XV are recovered in the simulations, with a reasonable rate of initiation (Fig. 9a). The simulations show that the tip accumulation of myosin-XV depletes after each aggregate forms, and each aggregate diminishes in amplitude as it approaches the filopodia base (Fig. 5 and Supplementary Figs. S1a, S3), both features in agreement with the observations (Fig. 3 and Supplementary Fig. S1b). The diminishing nature of the aggregates (as they approach the base) is evidence for motor treadmilling, as the model predicts: the very low influx of motors into the filopodia is not supplying enough motors to compensate for motors that leave the aggregate and processively walk to the tip.

Importantly, despite the good qualitative comparisons, the current model is minimal and naturally does not treat additional possible effects and processes that could be included in the future. Nevertheless, the good agreement strongly suggests that both myosin-XV and myosin-III generally detach from the actin bundle at the filopodia tip (*β* small, Fig. 5), possibly related to the release of their cargoes there. The observations suggest that *β* is time dependent and possibly stochastic. Functionality of the tip is therefore, not just a trigger but plays the role of selecting the patterns to be either pulses (trains) or TWs. The dynamics within the filopodia are probably affected by several additional sources, such as noise (thermal and active), temporal dependence of the chemical reactions at the tip (here *β* was treated as a fixed control parameter) and a relation between the actin polymerization rate at the tip and the density of motors (and their cargo) there. The relation between a continuum model and microscopic (discrete) models for these aggregates^22^ is another interesting issue for further investigation.

## Conclusion

The understanding of macroscopic transport properties by unconventional myosins inside cellular protrusions is of fundamental importance. While the process comprises complex feedback relations between the myosins fluxes, conversion transitions and the actin network, we show that the spatiotemporal behavior essentially emerges from self-organized principles in the form of traveling waves, pulses and pulse trains. The proposed underlying mechanism involves the cooperative (non-linear) interaction between motors, converting them from processive to non-processive. While the biochemical details may vary between motor types, we suggest that this mechanism leads to the general emergence of dynamic aggregates. The selection mechanism between the different spatio-temporal patterns is governed by the conditions at the protrusion base, and in particular at the tip. The tip is in fact the triggering source through which traveling waves or pulses are generated: for pulses the tip allows for repeated accumulation above the threshold, while TWs are spontaneously generated from an inherent instability (no threshold). Hereby, although the rearwards motion of myosin aggregates has been previously observed in filopodia, we point out and distinguish for the first time between distinct structures of these aggregates, specifically at their origin near the protrusion tip.

The issue of interactions between motors that coexist on the same filopodia is of importance as well. The specific case of both myosin-III and myosin-XV inside the same protrusion, as shown in Figs 1 and 9, is highly relevant for the study of stereocilia, where both these motors coexist^13^. The finite available volume between the actin and the surrounding membrane, and the finite size of the motors, implies that the local concentration of the motors along the bundle saturates at some maximal packing value. This result also sets an upper limit on the amplitudes of the emerged pulses and waves. If this limitation is too strong, the local concentration cannot reach the threshold needed to trigger the pulses and waves (Supplementary Fig. S5b), which is clearly not the regime inside filopodia. However, strong excluded volume interactions (see Supplementary), may occur inside stereocilia where the actin bundle is much larger than in filopodia, and so the available space is more constrained. Under such conditions we can expect strong entrainment between the motor species (Supplementary Fig. S6) and spatial segregation (Supplementary Fig. S7). Note that in Supplementary Fig. S7 we show that when static distributions (no pulses) form, the excluded volume interactions relegate the faster (more processive) motor to the tip, as is observed for the competition between myosin-XV and myosin-III in stereocilia^13^.

To mechanistically understand the empirical observations, we developed a simplified model that was constructed to keep fidelity to the most essential biological processes inside actin-based protrusions: (i) fluxes and interactions comprise dominant motor functionalities, i.e. processive motor motion, treadmilling actin and free diffusion, and (ii) boundary conditions account for realistic kinetic and flux properties. The theoretical framework allowed us also to experimentally revisit the dynamics of myosins-XV, with respect to the origin of traveling waves versus pulse trains and provide insights into the fundamental nature of molecular motor transport. In addition, it follows that further model extensions toward more realistic comparisons with experiments should include dynamics (deterministic and stochastic) of the conditions at the tip, i.e. temporal changes in *β*. Moreover, the distinction between transport modes exploits a reaction-diffusion-advection framework constrained by mass conservation, which is a methodology that may be applicable to a variety of non-equilibrium systems.

Finally, current model provides several testable predictions that await future experiments: we anticipate a strong dependence of the dynamics of motor aggregates on the rate of actin polymerization *v*_*b*_, as compared to the processive velocity of the motors. In addition, we conjecture that the same mechanism should have influence on the length of the filopodia. Both of these features (Figs 5 and 6) may be amenable to experimental manipulations.

## Methods

### Expression plasmids

The mCherry-tagged myosin-IIIa delta-kinase construct used was previously generated in our laboratory (Salles *et al*., 2009). We used the delta-kinase version of the myosin-IIIa because the presence of the kinase domain inhibits the activity of this class myosin (Salles *et al*., 2009). The construct encoding for eGFP-tagged myoXVa (NM_182698, product size 2,306 amino acids) was a kind gift from Dr TB Friedman (NIDCD).

### COS7 cell transfection

COS7 cells were plated in 30 mm coverslip-bottom cell culture dishes (MatTek Corporation) and maintained at 37 °C in DMEM supplemented with 10% FBS (Hyclone). Cultures were transfected using Lipofectamine LTX (Life Technologies), and imaged 24 h after transfection.

### Live imaging

COS7 cells were imaged using a Nikon TiE inverted fluorescence microscope, outfitted with a spinning disk confocal scan head (Yokogawa), 100 × 1.49 N.A. objective, and Andor DU-888 EM-CCD. Coverslip-bottom dishes were placed in a stage top incubator with CO2 concentration maintained at 5%, and temperature at 37C under humidified conditions for the duration of the imaging experiments. Multichannel images were acquired at 500 ms exposures per channel, every 5 seconds for an average of 10 min per dataset. Acquisition of all datasets was managed through NIS-Elements software.

### Kymographs

Kymographs of the fluorescence-tagged myosin motors were generated in ImageJ (NIH). The changes in fluorescence intensity of the moving myosin cluster were determined by integrating the fluorescence intensity of a 9 pixel square region of interest overlaying the cluster for each frame. Intensity Profile Analysis The profile of intensity along filopodia through time was analyzed using NIS-Elements by aligning the dataset relative to the myosin-III-mCherry-rich tip to both correct for drift and anchor the filopodium for subsequent analysis of flow relative to the tip. A maximum intensity projection through time was generated to verify that the length of the filopodia did not shift laterally during the course of the analysis. A two-pixel thick profile was traced along the length of the filopodium such that it overlaid the centerline, and as such, the path of retrograde flow. Data reflecting the intensity values of each pixel along the profile for both channels through time was exported and plotted in Igor Pro (Wavemetrics). Representative graphs were chosen to highlight the course of accumulation and propagation of myosin-XVa-eGFP clusters.

### Nonlinear equations for the motor dynamics inside the protrusion

Accounting for the biological transport fluxes and the kinetic rates (described in Section 1.2), the continuum one-dimensional equations for the three motor concentrations, i.e., (i) left propagating (*m*_*w*_) with velocity *v*_*w*_ (ii) right propagating (*m*_*b*_) with velocity *v*_*b*_ and (iii) free diffusing *m*_*f*_, read:


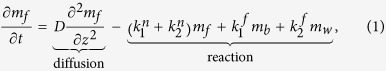



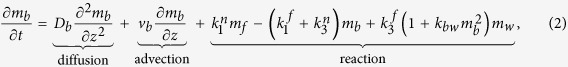



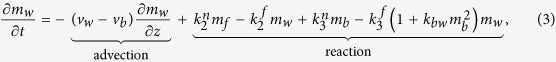


where 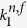, 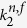 and 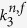 are the respective on/off rates of the transition from free to stalled, free to processive and stalled to processive, *k*_*bw*_ is the coefficient for the non-linear conversion term of processive motors to stalled. The transport properties of the concentrations (number of molecules per unit length) are estimated as: (i) *m*_*f*_ freely diffusing motors with diffusion coefficient *D* ~ 0.1 *μm*^2^/*sec*; and (ii) processive motors walking along the filaments towards the tip, *m*_*w*_, with relative velocity *v*_*w*_ ~ 0.2–1 *μm*/*sec* and no diffusion (*D*_*w*_ = 0). Stalled motors (*m*_*b*_) that are attached to an actin filament do not complete their mechanochemical cycle, and are therefore carried towards the protrusion base at the treadmilling velocity of the actin filaments (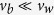). However even if stalled, they may still be able to perform random forward and backward steps^17^ which result in a small effective diffusion along the actin filaments, i.e. 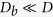.

The stalling process can be described in a series expansion in powers of 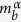 (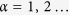), where only *α* ≥ 2 yields nonlinear self organization, such as a finite wavenumber Hopf instability to TWs^27^,^28^. Thus, for simplicity, we consider only the quadratic term, *α* = 2 (for *α* = 1 the stalled motors uniformly fill the length of the protrusion, similar to the saturation effect of excluded volume interactions, see Supplementary). In fact quadratic terms are frequently used in chemical autocatalytic reactions, such as the Brusselator model for the oscillatory Belousov-Zhabotinsky chemical reaction^31^.

The model presented above makes another simplification, by restricting the distinction between active and inactive forms of the motors only when actin-bound, while losing this distinction when freely diffusing. A complete treatment would involve *m*_*f,on/off*_ for the two forms of freely diffusing motors. However, the simplification used here pertains to the case where the activity state of the motor becomes lost on a fast time-scale when it is not actin-bound. Note that excluded volume effects are not included in Eqs. 1, 2, 3, since we wish to investigate the effects of the non-linear stalling interactions in the simplest possible system (see Supplementary for the effects of excluded volume, Fig. S5). Furthermore, an alternative form of non-linear interactions, whereby processive motors detach upon interaction with stalled motors, does not lead to oscillatory behavior (Supplementary Fig. S2).

### Dynamics at the protrusion limits: boundary conditions

Eqs. 1, 2, 3 are supplemented with boundary conditions (BC) that reflect realistic properties of motors once they reach respectively, the protrusion tip or base, see Fig. 4. At the protrusion base (*z* = 0), the concentration of freely diffusing myosins is fixed and determined by the concentration in the cell cytoplasm, while the processive myosins are attributed to have a fixed influx and the stalled motors are convected out from the protrusion:


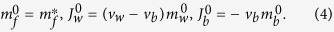


At the tip (*z* = *h*), since the protrusion is closed there must be an overall zero flux condition:


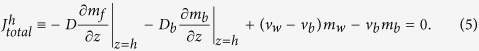


Furthermore, the processive myosins may either be released from the actin and become freely diffusing, become stalled, or some combination between these two limiting cases. Consequently, the BC are described by a fraction (*β*) that controls the transition of the processive motors that become stalled at the tip and respectively the flux of free myosins:


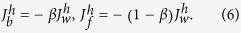


Here *β* = 1 corresponds to pure transition between the counter propagating subsets *m*_*w*_ → *m*_*b*_, while *β* = 0 denotes a pure transition to freely diffusing *m*_*w*_ → *m*_*f*_. Notably, *β* = 1 is consistent with the vanishing of *J*_*total*_ for uniform distributions, see Eq. 5.

### Range of parameter values

Throughout the analysis, we consider the parameters to have these typical values: *v*_*w*_ = 0.1–1 *μ*m/sec, *v*_*b*_ = 0.05–0.001 *μ*m/sec, *D* = 0.1 *μ*m^2^/sec, *D*_*b*_ = 0.0005 *μ*m^2^/sec, 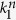 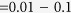
sec^−1^, 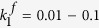
sec^−1^, 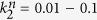
sec^−1^, 
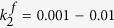
sec^−1^, 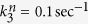, 
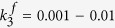
sec^−1^, and *k*_*bw*_ = 0.1–10 *μ*m^2^ (densities are per unit length).

Rates are very difficult to obtain via empirical measurements, and furthermore, as we noted above, in our model they constitute a coarse-grained version of a more complex set of chemical reactions. Highly processive motors, such as myosin-X and myosin-XV, should have a large ratio of: 
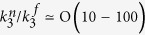
 (estimated from single motor tracking^17^), and the high affinity of the motors to the actin means that we expect: 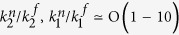.

### Uniform solutions and linear stability analysis

To analyze the distinct solutions of the model equations and their properties we start by looking at uniform steady-state solutions to Eqs. 1, 2, 3, 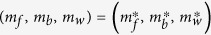. Uniform solutions are obtained by setting the partial derivatives to zero, i.e. 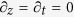:


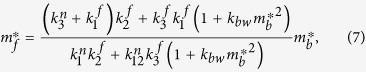



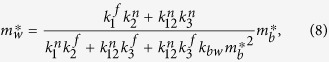


where 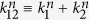. Due to mass conservation, Eqs. 7,8 show that one of the fields can be set arbitrarily (Fig. 7a), i.e. an inherited degeneracy that is similar to systems that obey continuity, such as thin fluid films described by Cahn-Hilliard models^32^).

Since the protrusion is an open system, degeneracy of uniform states can be removed in two ways, both are related explicitly or implicitly to BC. The first corresponds to the intracellular properties of the cell itself, which determine the density of tip-directed and free motors at the protrusion base. In the mathematical context, it corresponds to low values for the uniform solution at the protrusion base (*z* = 0), which we denote as lower uniform state: 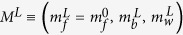. The second way is due to the overall zero-flux condition within the protrusion (Eq. 5), which forces the uniform fields to obey the relation:


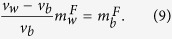


In what follows, we refer to the uniform state that results from the zero flux constrain as upper uniform state: 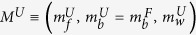. Since *v*_*b*_ 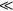 *v*_*w*_, the value of *m*_*b*_ is rather large (Fig. 7a), in comparison to the low concentrations of the free motors (and respectively all other motors) in the cytoplasm.

To understand the sensitivity of the uniform states to perturbations, we perform a linear analysis. On infinite domains, the temporal evolution is obtained by deriving the growth rates that are associated with weak spatially periodic perturbations about the uniform state:


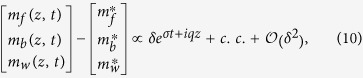


where 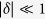, *σ* is the dispersion relation, *q* > 0 is the wavenumber, and *c*.*c*. stands for complex conjugate.

We are interested in two limiting cases, uniform solutions *M*^*L*^ and *M*^*U*^. For *M*^*U*^ solutions the degeneracy is removed only by initial conditions or boundary conditions while the *M*^*L*^ states are determined at the protrusion base the values are fixed by the value of the freely diffusing motors in the cytoplasm *m*_*f*,0_. Since the BC at *z* = 0 is fixed by the cytoplasm, the pattern selection depends on the conversion ratio (or accumulation) of the stalled motor density at the tip, *β*. The conversion ratio *β* = 1 corresponds to the mass conservation according to Eq. 9 and in turn determines the approach to *M*^*U*^. Near the tip, we use the zero-flux condition to fix the relation between the walking and stalled motors, 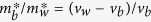, which fixes 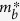:


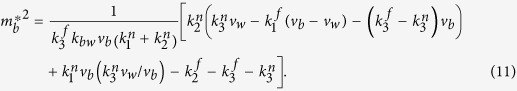


We find that the maximum of the graph of 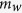 as a function of 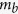 is shifted to larger values according to:


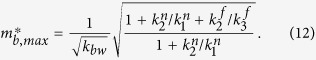


Substitution of Eq. (10) into Eqs. (1, 2, 3) yields three characteristic dispersion relations once *m*_*b*_ is increased above a critical value, 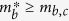: two with negative growth rates, 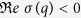 for all *q*, and one which exhibits a finite wavenumber instability to TWs where the real part becomes positive for a finite band of wavevectors 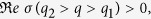 *m* *σ* (*q*) > 0, as numerically shown in Fig. 7b,c. The latter dispersion relation also admits a soft mode, 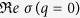 = *m* *σ* (*q* = 0) = 0 which corresponds to the translational symmetry of the uniform states due to mass conservation. Below the critical value, 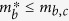 the uniform *M*^*L*^ states are linearly stable but unstable to large amplitude perturbations while above the onset the uniform state is unstable however, weak perturbations develop to large amplitude TWs. Both behaviors are features of the so-called *sub-critical* instability type^29^,^33^. Mathematically, it means that the branch of TWs bifurcates towards the stable uniform state and is initially unstable^28^. The group velocity of the linear perturbations is larger than the convection velocity *v*_*b*_ for long wavelengths (*q* → 0, Fig. 7c), but with increasing wavevector this velocity decreases monotonously, and is approaching *v*_*b*_ from *above* for large *q*. Therefore, the propagation velocity is necessarily smaller than the advection due to the treadmilling actin (*v*_*b*_), for large wavevectors. This mix of fast and slow wavelengths, results in the behavior which we demonstrate in Fig. 7d,e: the instability is not washed away from its initiation site as in a simple convective instability, but the amplitude of the oscillations at the initiation point decays exponentially with time.

Following the results from linear stability analysis, we choose *M*^*U*^ to be in the unstable to TWs regime (see Fig. 7b,c); especially for the biologically relevant regime of small values of 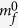. Through numerical simulations of Eq. 1, 2, 3, we show in Fig. 8 that variation of *β* between 0 to 1 is responsible for the generation of pulses and TWs, respectively.

## Additional Information

**How to cite this article**: Yochelis, A. *et al*. self-organization of waves and pulse trains by molecular motors in cellular protrusions. *Sci. Rep*. **5**, 13521; doi: 10.1038/srep13521 (2015).

## Supplementary Material

Supplementary Information

Supplementary Movie S1
