# Supplementary material for: Self-organization of waves and pulse trains by molecular motors in cellular protrusions
Source: Sci Rep. 2015 Sep 3;5:13521. doi: 10.1038/srep13521 (PMC4558574; doi:10.1038/srep13521)
Supplement: Supplementary Information [file srep13521-s1.pdf]

Supplementary Information:

The origin and self-organization of waves and pulse trains by molecular motors in cellular protrusions

A. Yochelis,[1] S. Ebrahim,[2] B. Millis,[2] R. Cui,[2] B. Kachar,[2] M. Naoz,[3] and N. S. Gov[3]

[1] *Department of Solar Energy and Environmental Physics, Swiss Institute for Dryland Environmental and Energy Research, Blaustein Institutes for Desert Research (BIDR), Ben-Gurion University of the Negev, Sede Boqer Campus, Midreshet Ben-Gurion 84990, Israel**

[2] *Laboratory of Cell Structure and Dynamics, NIDCD, National Institutes of Health, 50 South Drive, Bethesda, MD 20892-8027, U.S.A.*

[3] *Department of Chemical Physics, Weizmann Institute of Science, P.O.B. 26, Rehovot, Israel 76100*

* yochelis@bgu.ac.il

CAPTION FOR SUPPORTING MOVIE 1

Confocal time lapse imaging of filopodia induced by transfection of COS7 cells with Myo3adK-mCherry (red) and MyoXVa-eGFP (green).

DECAY OF THE MYOSIN-XV PULSES

In Fig. S1 we show the decay of a single myosin-XV pulse as it propagates to the protrusion base: (a) simulation and (b) experiment.

INTERACTING MOTORS FALL-OFF

We investigate an alternative scenario, where the non-linear interaction (collisions) between the walking and stalled myosins result in the freeing of the walking myosins, i.e. they fall-off the actin and become freely diffusing. We therefore use the *same* non-linear interaction term that appears in Eqs.2,3, namely

$$\frac{\partial m_f}{\partial t} = D\frac{\partial^2 m_f}{\partial x^2} - k_1^n m_f + k_1^f m_b - k_2^n m_f + (k_2^f + k_3^f k_{bw} m_b^2) m_w \tag{S1}$$

$$\frac{\partial m_b}{\partial t} = v_b \frac{\partial m_b}{\partial x} + k_1^n m_f - k_1^f m_b - k_3^n m_b + k_3^f m_w \tag{S2}$$

$$\frac{\partial m_w}{\partial t} = -v_w \frac{\partial m_w}{\partial x} + k_2^n m_f - k_2^f m_w + k_3^n m_b - k_3^f (1 + k_{bw} m_b^2) m_w \tag{S3}$$

We find that in such a case no oscillations occur in the system and the effect of the collision term in this case is to weaken the localization of myosins to the tip in a similar manner to a saturation effect (Fig. S2). This indicates that in order to have oscillation in the system the walking myosins must be transformed by collisions into stalled myosins and not free myosins.

BOUNDARY CONDITION OF $\beta = 0$

In Fig. S3 we show the space-time plot for myosin-XV-like parameters (as in Fig. 5d), for the boundary-condition of $\beta = 0$. The first pulses that form at the tip decay fast as they propagate towards the protrusion base (for low values of M^L), but over time the overall background values of the motors along the protrusion length increase and the pulses decay more slowly, eventually reaching the filopodia base.

BOUNDARY CONDITION OF $\beta = 1$

In Fig. S4 we show the simulation for myosin-III-like parameters (as in Fig. 7e), for the boundary-condition of $\beta = 1$. We find that the filopodia tends to fill up with motors, unlike the observations (Figs. 1-3, 7), indicating that the BC in the filopodia corresponds to smaller values of β.

STERIC (EXCLUDED-VOLUME) INTERACTIONS BETWEEN MOTORS

In order to take into account the limited space on the actin bundle along the protrusion, we may introduce an upper bound on the concentration of actin-bound motors that can occupy per unit length, ν. We implement this into our continuum model by multiplying the ”on”-rate from the free component to the actin-bound components by a cut-off function, as well as the terms for the current of processive motors. The cut-off function is simpley

$$\mathcal{C} = 1 - \frac{1}{\nu} \sum_i (m_{b,i} + m_{w,i}) \tag{S4}$$

where the summation i goes over all the different species of motors that co-inhabit the actin bundle, and the constraint: $\mathcal{C} \geq 0$. Another practical way of implementing this cut-off in a continuous manner is to use an exponential form

$$\mathcal{C} = \exp\left[-\frac{1}{\nu}\sum_i (m_{b,i} + m_{w,i})\right] \tag{S5}$$

which is the way we used in the simulations (both forms give very similar results).

The equations of motion are therefore modified compared to Eqs. 1

$$\frac{\partial m_f}{\partial t} = D\frac{\partial^2 m_f}{\partial z^2} - (k_1^n + k_2^n)\,\mathcal{C}m_f + k_1^f m_b + k_2^f m_w \tag{S6}$$

$$\frac{\partial m_b}{\partial t} = D_b\frac{\partial^2 m_b}{\partial z^2} + v_b\frac{\partial m_b}{\partial z} + k_1^n\mathcal{C}m_f - \left(k_1^f + k_3^n\right) m_b + k_3^f\left(1 + k_{bw}m_b^2\right) m_w \tag{S7}$$

$$\frac{\partial m_w}{\partial t} = -\left(v_w\mathcal{C} - v_b\right)\frac{\partial m_w}{\partial z} + k_2^n\mathcal{C}m_f - k_2^f m_w + k_3^n m_b - k_3^f\left(1 + k_{bw}m_b^2\right) m_w \tag{S8}$$

The saturation effect is shown for the case of a single specie of myosin in Fig. S5. We used here the parameters that seem to capture the behavior of the myosin-XV observed in the filopodia experiments: $v_w = 600$nm/sec and $v_b =$ 16nm/sec, while the other parameters are: $D = 0.1\mu\text{m}^2/\text{sec}$, $D_b = 0.0005\mu\text{m}^2/\text{sec}$, $k_1^n = 0.01\text{sec}^{-1}$, $k_1^f = 0.01\text{sec}^{-1}$, $k_2^n = 0.03\text{sec}^{-1}$, $k_2^f = 0.005\text{sec}^{-1}$, $k_3^n = 0.1\text{sec}^{-1}$, $k_3^f = 0.01\text{sec}^{-1}$, $k_{bw} = 2\mu\text{m}^2$ and $m_f^0 = 0.05\mu\text{m}^{-1}$.

As we expect, we find that when the cut-off is very large (Fig. S5a) the pulses are the same as without any cut-off, while as it decreases the pulses become reduced in amplitude (Fig. S5b). Since the amplitude is smaller and the width is largely unaffected, the frequency of the pulses increase (to conserve the overall current). When the cut-off is far enough below the uniform zero-flux concentration of the stalled motors (M^U), we find that the pulses are inhibited (Fig. S5c).

In Fig. S6 we plot the dynamics of two species of interacting motors, where the myosin-XV has $v_w = 600$nm/sec, while the myosin-III has $v_w = 75$nm/sec, with all the other parameters taken as equal for both motors. When the cut-off parameter is large (Fig. S6a) there is weak entrainment of the myosin-III by the myosin-XV. Myosin-III aggregates form at a very late time, and lower frequency and lower amplitude compared to the myosin-XV aggregates. The accumulation of myosin-III at the tip is only slightly affected by the release of each myosin-III pulse. For a smaller cut-off value (Fig. S6b) the interaction increases, and the pulses of myosin-III start earlier and with larger amplitude, the tip concentration of myosin-III now oscillates strongly. At very low cut-off value the pulses of myosin-XV have lower amplitude, but with increased frequency (as in Fig. S5b), with the myosin-III pulses fully entrained and with similar characteristics (Fig. S6c).

FILOPODIA VS STEREOCILIA

In the stereocilia, the rates of actin polymerization are 2-3 orders of magnitude smaller than in the filopodia [1]. We first check the effects of this decrease of the polymerization rate on the distribution of the motors (myosin-XV, III) when the interaction between them is negligible ($\nu = 700, 1200$). We find that unlike in filopodia (Fig. S6a,b), the myosin-XV does not form pulses (Fig. S7a), but has a static steady-state distribution near the tip, as does the myosin-III.

When the steric constraint is very strong, $\nu = 10$ (Fig. S7b), we find that the motors segregate, with the myosin-XV localize at the tip, and myosin-III expelled from the tip and extends over most of the protrusion length towards its base. This pattern is similar to the pattern observed in mature stereocilia [1], therefore suggesting the following hypothesis:

- The steric constraint inside the stereocilia is much more stringent compared to the filopodia. This may arise from the crystal-like order of the actin filaments inside the stereocilia, compared to the more disordered organization inside the filopodia [2].

- The strong localization of the myosin-XV to the tip is partly due to the concentration of myosin-III that is "piling up" below it on the stereocilia. The interactions between the myosin species is therefore seen to play a role in their final steady-state distribution, in this system.

[1] Rzadzinska, A. K., Schneider, M. E., Davies, C., Riordan, G. P. & Kachar, B. An actin molecular treadmill and myosins maintain stereocilia functional architecture and self-renewal. *J. Cell Biol.* **164**, 887–897 (2004).
[2] Medalia, O. *et al.* Organization of actin networks in intact filopodia. *Curr. Biol.* **17**, 79–84 (2007).

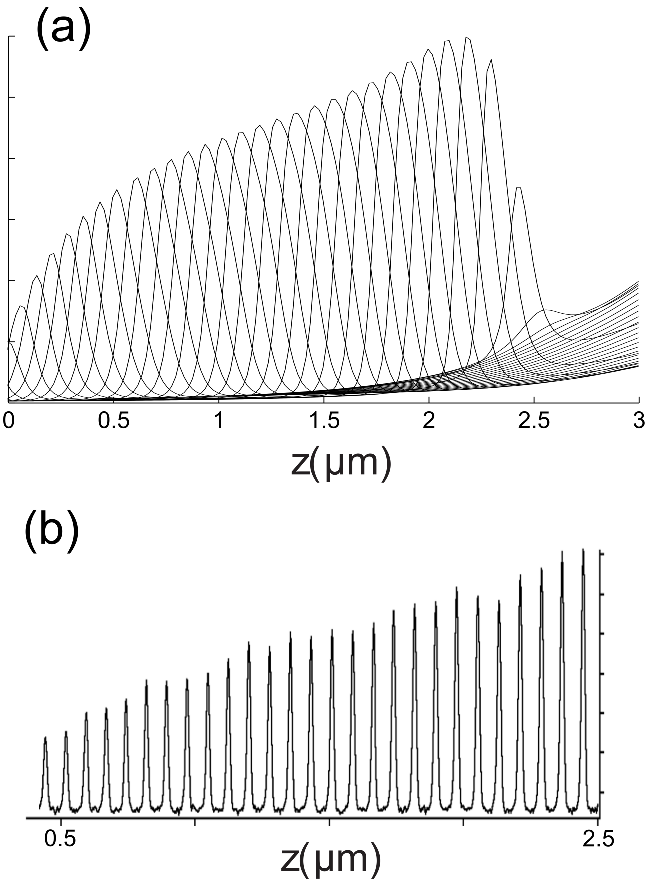

FIG. S1. (a) Calculated decay of a single pulse, using myosin-XV-like parameters (as in Fig. 6). (b) Experimental myosin-XV concentration profile of a single aggregate, overlayed at different times, as it propagates to the protrusion base, and decays.

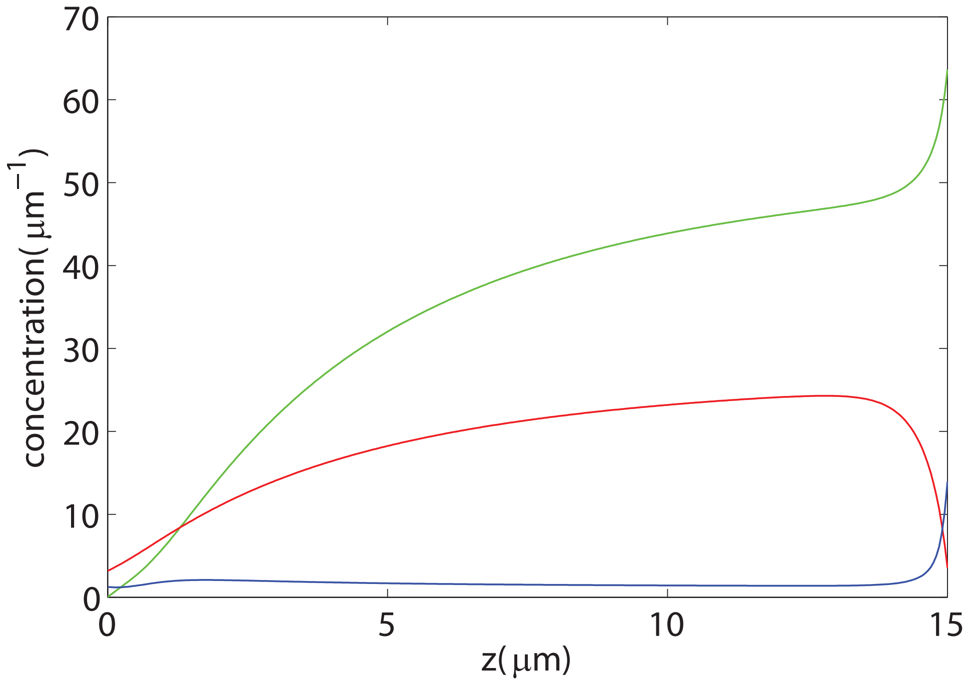

FIG. S2. A numerical integration of Eqs. S1-S3 with the parameter values given in the text. We see that in this case no oscillation are present and the concentration profile is smooth and much flatter than in the linear case, i.e, the localization effect is significantly weaker. The green, red and blue lines are the free, stalled and walking myosins concentration respectively.

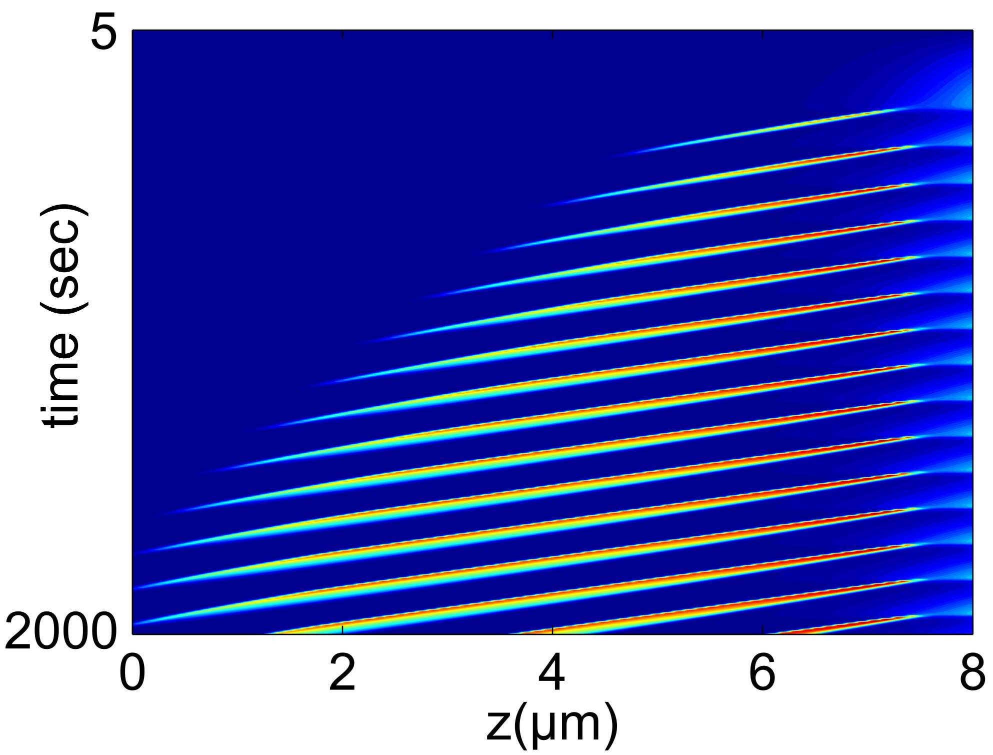

FIG. S3. Space-time plot of the simulation results for total concentration of myosin-XV, using parameters as in Fig. 5.

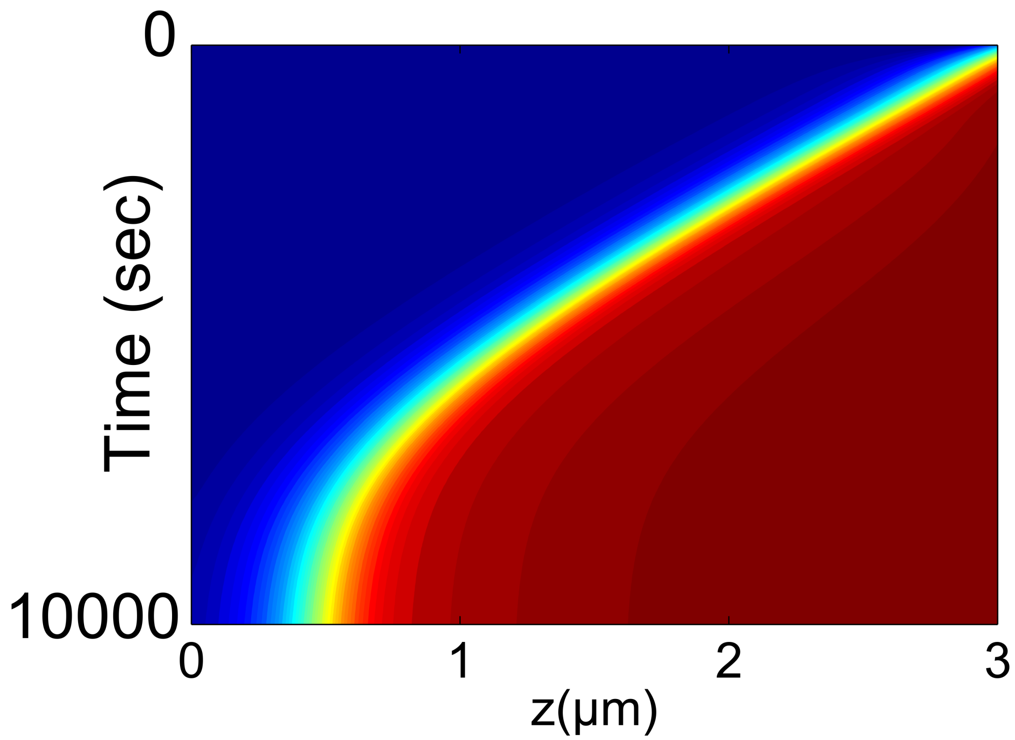

FIG. S4. Simulation results for myosin-III-like parameters (as in Fig. 7e), except for the value: $\beta = 1$.

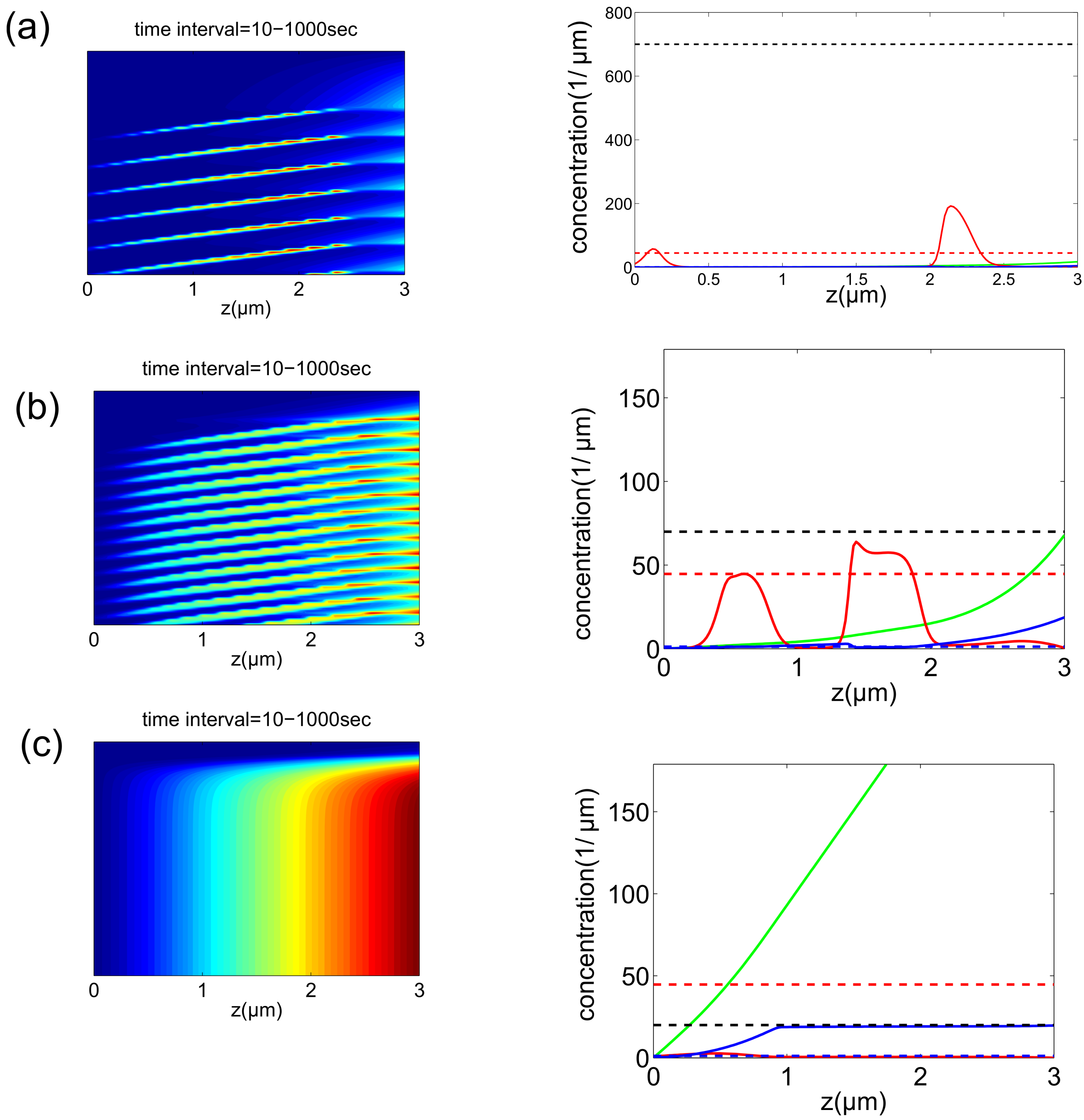

FIG. S5. A numerical integration of Eqs. S6-S8 with the parameter values given in the text (for myosin-XV-like motors), for different strength of the steric cut-off parameter $\nu = 700, 70, 20$ (from top to bottom). Space-time plots of the total myosin concentration are shown in the left panels, and a snap-shot from the final time in the right panels. The steric cut-off parameter is indicated by the horizontal dashed black line. The green, red and blue lines are the free, stalled and processive myosins concentration respectively, and the horizontal dashed red line indicates the uniform zero-flux concentration of the stalled motors (M^U).

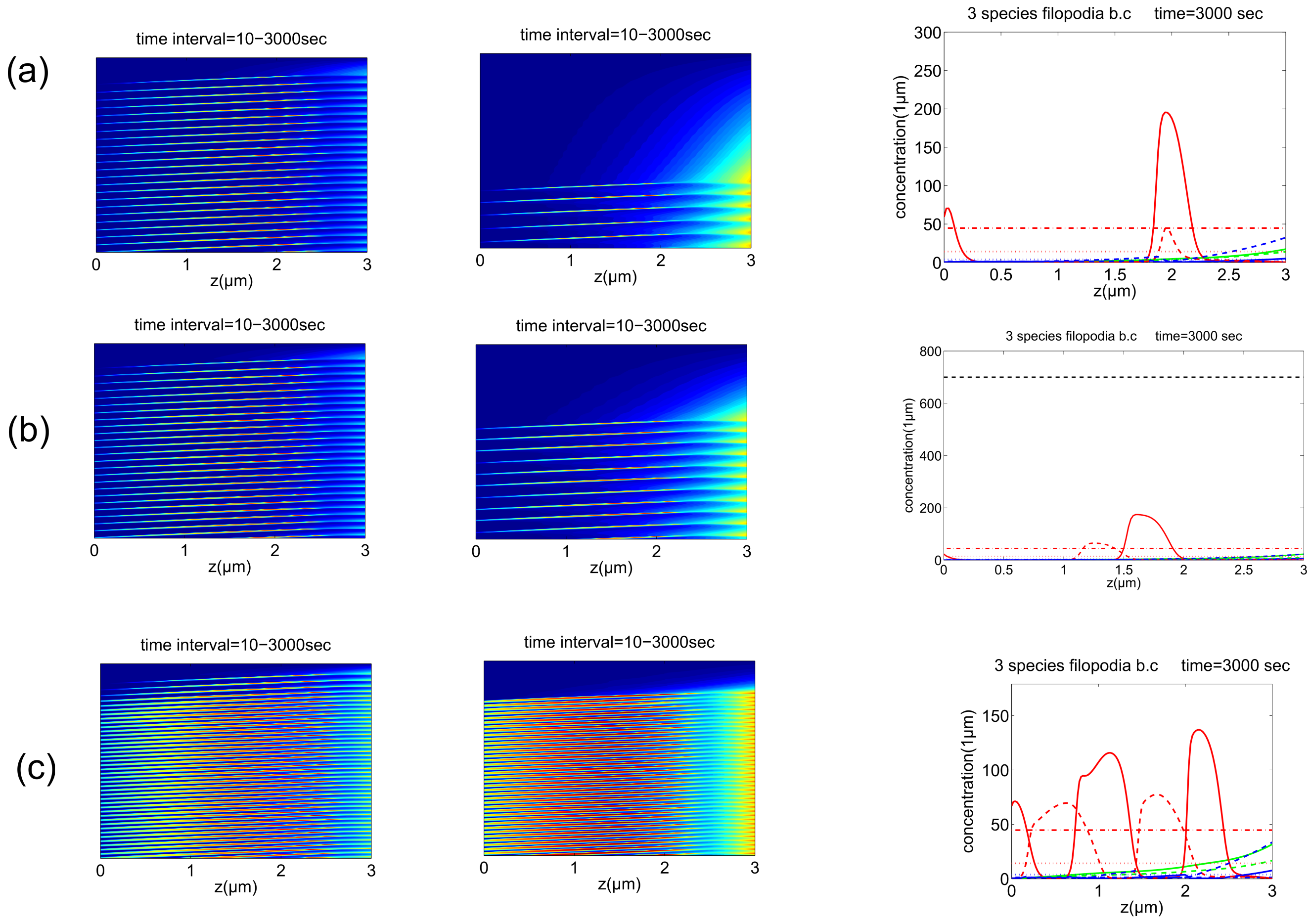

FIG. S6. A numerical integration of Eqs. S6-S8 with the parameter values given in the text, for different strength of the steric cut-off parameter $\nu = 1200, 700, 200$ (from top to bottom). Space-time plots of the total myosin concentrations are shown for the two motor species in the left panels, and a snap-shot from the final time in the right panels. The green, red and blue full (dashed) lines are the free, stalled and processive myosins concentration respectively, for the myosin-XV (myosin-III) motor. The horizontal dashed-dot (dotted) red line indicates the uniform zero-flux concentration of the stalled motors (M^U) of the myosin-XV (myosin-III) specie.

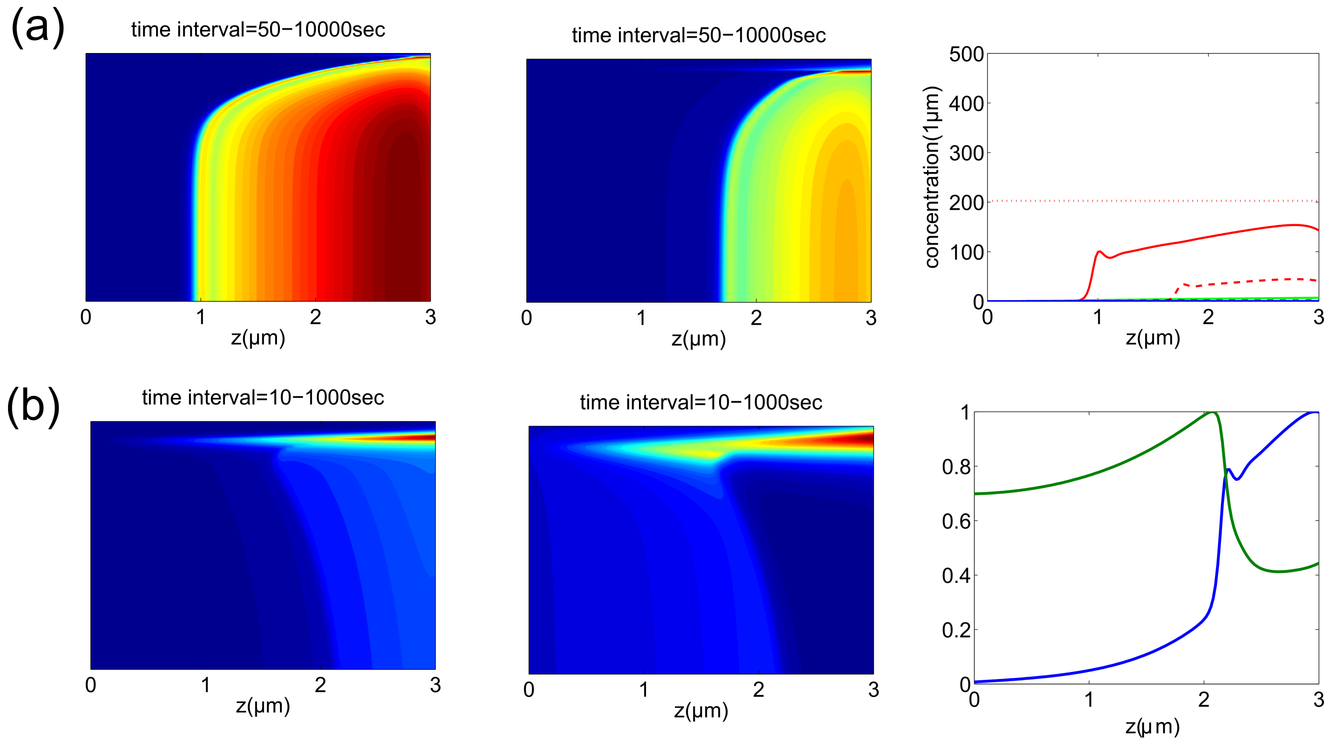

FIG. S7. A numerical integration of Eqs. S6-S8 with the parameter values given in the text, for different strength of the steric cut-off parameter $\nu = 700, 10$ (from top to bottom). Space-time plots for the total concentrations of two motors are shown in the left panels, and a snap-shot from the final time in the right panels. in (b) the rightmost panel shows the distribution of the total normalized concentrations (normalized by the maximum value) for each motor. Color scheme of the lines as in Fig.S6.
